# Sub-National Targeting of Seasonal Malaria Chemoprevention in the Sahelian Countries of the Nouakchott Initiative

**DOI:** 10.1371/journal.pone.0136919

**Published:** 2015-08-31

**Authors:** Abdisalan Mohamed Noor, Eliud Kibuchi, Bernard Mitto, Drissa Coulibaly, Ogobara K. Doumbo, Robert W. Snow

**Affiliations:** 1 INFORM (Information for Malaria – www.inform-malaria.org), Spatial Health Metrics Group, Kenya Medical Research Institute/Wellcome Trust Research Programme, Nairobi, Kenya; 2 Centre for Tropical Medicine and Global Health, Nuffield Department of Clinical Medicine, University of Oxford, Oxford, United Kingdom; 3 Malaria Research and Training Center, University of Sciences, Techniques and Technologies, Bamako, Mali; London School of Hygiene and Tropical Medicine, UNITED KINGDOM

## Abstract

**Background:**

Seasonal malaria chemoprevention (SMC) has been shown to be highly efficacious against clinical malaria in areas where transmission is acutely seasonal. SMC targeting depends on a complex interplay of climate, malaria transmission and population distribution. In this study a spatial decision support framework was developed to identify health districts suitable for the targeting of SMC across seven Sahelian countries and northern states of Nigeria that are members of the Nouakchott Initiative.

**Methods:**

A spatially explicit decision support framework that links information on seasonality, age-structured population, urbanization, malaria endemicity and the length of transmission season was developed to inform SMC targeting in health districts. Thresholds of seasonality, population and receptive risks were defined to delineate SMC suitable health districts and define the age range of children for targeting. Numbers of children were then computed for the period 2015–2020 in SMC districts. For 2015, this was combined with maps of length of malaria transmission seasons and WHO recommended treatment regimen to quantify the number of tablets required across the SMC health districts.

**Results:**

A total of 597 Sahelian health districts were mapped, out of which 478 (80.1%) were considered suitable for SMC based on seasonality and endemicity thresholds. These districts had an estimated 119.8 million (85%) of the total population in 2015. In the six years from 2015–2020, it is estimated that a total of 158 million children 3m to <5 years, 121 million of whom were in rural areas, will need SMC to achieve universal coverage in the Sahel. If the upper age limit of SMC targeted children was increased to <10 years in low transmission districts, a total 177 million overall, of whom 135 million were rural children, will require chemoprevention in 2015–2020. In 2015 alone, an estimated 49–72 million SP tablets and 148–217 million AQ tablets will be needed to cover all or rural children respectively under the different scenarios of upper age limits.

**Conclusions:**

Our proposed framework provides a standardised approach to support targeting and scale up of SMC by the countries of the Nouakchott Initiative. Our analysis suggests that the vast majority of the population in this region are likely to benefit from SMC and substantial resources will be required to reach universal coverage each year.

## Introduction

Seasonally targeted intermittent preventive treatment of malaria in children, also known as seasonal malaria chemoprevention (SMC), has been shown, under trial conditions, to prevent approximately 75% of clinical malaria episodes, including severe malaria, in areas where transmission is concentrated within a few months of the year [1,2]. In February 2012, the World Health Organization (WHO) approved a recommendation for the use of sulphadoxine-pyrimethamine plus amodiaquine (SP+AQ) for SMC in children aged 3 months to below 5 years administered at monthly intervals during the transmission season, principally in the Sahelian region of Africa [3]. In May 2013, the Nouakchott Initiative was signed by the governments of The Gambia, Chad, Mali, Mauritania, Niger and Senegal to accelerate and coordinate the fight against malaria in these six countries [4]. This was later expanded to include Burkina Faso and Nigeria, with the SMC focus in the latter on the nine northern States.

SMC targeting depends on a complex interplay of seasonality, the length of the transmission seasons, endemicity, population distribution and urbanisation. Where routine health systems remain weak, the most reliable sources of malaria risk are those that predict the intensity of *P*. *falciparum* transmission [5]. However, their use for defining endemicity thresholds for SMC is not straightforward. Previous analysis on defining SMC target population relied on recent predictions of malaria transmission intensity to define endemicity thresholds for SMC suitable areas and produced national estimates of target populations [6]. The use of the most recent malaria risk maps to define endemicity thresholds for SMC suitability, however, may exclude areas that have acute malaria seasonality but have transitioned to low levels of transmission.

Where transmission potential in such areas is high due to the continued presence of efficient vectors and large numbers of asymptomatic human hosts, SMC is critical to sustain low disease incidence and maps of receptive risks are better suited to defining endemicity thresholds for SMC. Conversely, as transmission declines to very low levels, the age pattern and the clinical burden posed by *P*. *falciparum* infection changes, with older children at greater risk [7,8]. Increasing the potential maximal benefit may require expanding SMC to cover older children and recent malaria risk maps should be used for such a decision. To support quantification of resources for sub-national scale up of SMC, suitability must also be resolved to health decision-making units, and within these units, information on population by residence (urban vs rural) and the number of months of malaria transmission are required.

In this study, we develop a spatial decision support framework that captures the interplay of seasonality, age-structured population, urbanization, endemicity and the length of transmission season to inform SMC targeting in Burkina Faso, Chad, Gambia, Mali, Mauritania, Niger, Senegal and nine northern states of Nigeria (Bauchi, Borno, Jigawa, Kano, Katsina, Kebi, Sokoto, Yobe and Zamfara). Layers of information are assembled and analysed to empirically quantify each element of the SMC spatial decision support framework. SMC health districts are defined and the numbers of target children by age range are estimated for the period 2015–2020, coinciding with the baseline and the first milestone years of the new WHO Global Technical Strategy for malaria [9]. For illustration, this information is then used to quantify the amount of tablets required for 2015 across the SMC health districts.

## Materials and Methods

### A spatial decision support framework for SMC targeting

**Fig 1:**
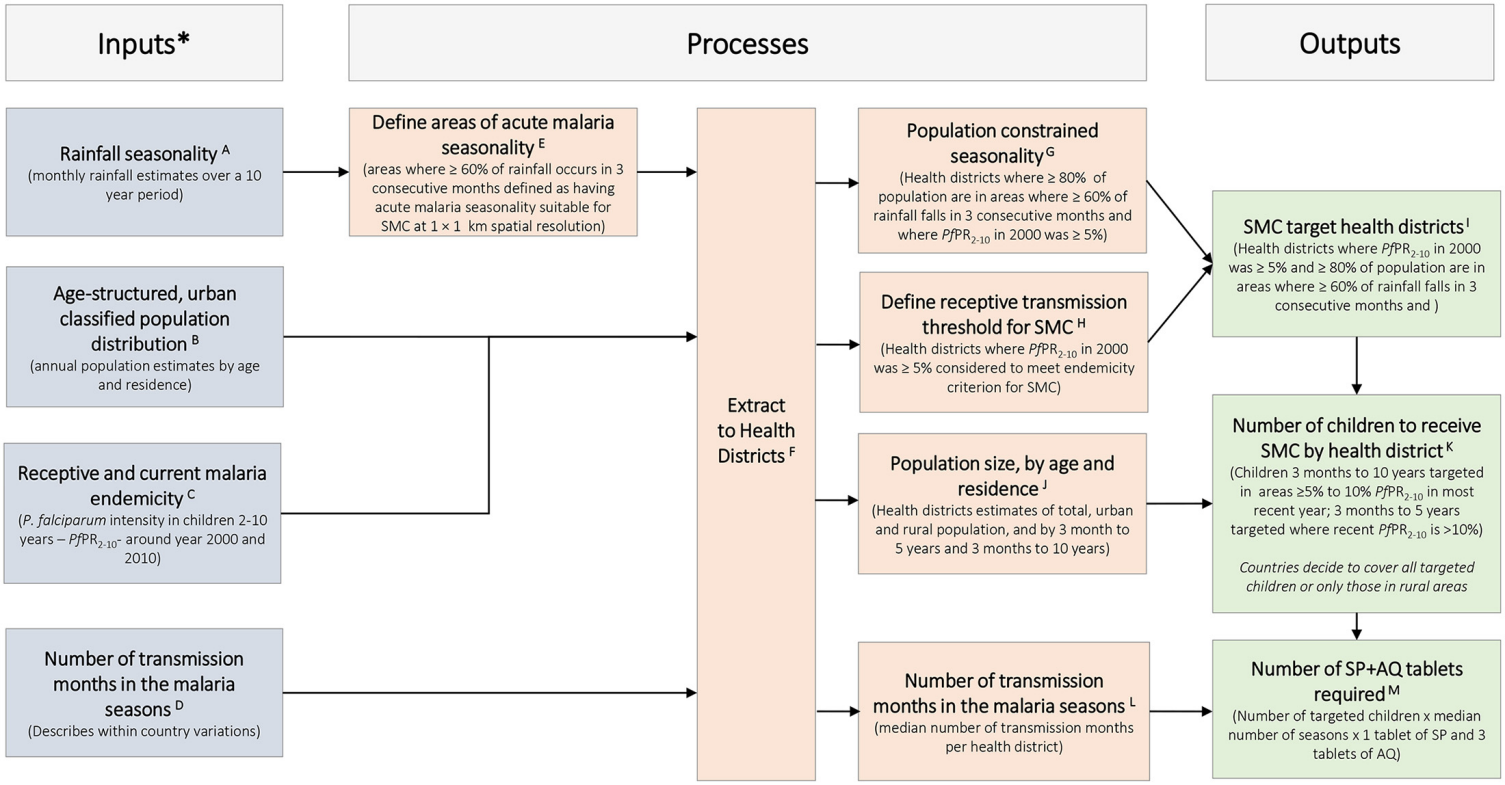
A spatial decision support framework for identifying areas suitable for seasonal chemoprevention and quantifying the size of the population of target children and the amount of the required antimalarial tablets. For additional details of the definition of inputs, processes and outputs see the S1 File.

The spatial decision support framework for SMC targeting that we propose in this study (Fig 1) is different to previous work [6] in several important aspects. A 2000 *P*. *falciparum* malaria risk map [5], an approximate measure of receptive risks prior to the large scale up of malaria interventions, is used to define endemicity thresholds for SMC suitability. A 2010 *P*. *falciparum* malaria risk map [5] is used to identify areas where the option of increasing the age class of target children from 3 months to below 5 years to up to below 10 years may confer greater benefit from SMC. We resolve Information to current health decision-making units, known as health districts, and age-structured populations within their boundaries are classified into urban and rural. The numbers of children that require SMC are estimated for the period 2015–2020 using population projections. Finally, median number of malaria transmission months is computed per health district to allow for the quantification of the amount of SP and AQ tablets required to achieve universal coverage in SMC districts in 2015.

### Assembly of input spatial data and layers

Detailed descriptions of the assembly of spatial layers of information are provided in the footnotes for Fig 1 and the accompanying S1 File. In brief, information from UN approved second-level administrative boundary demarcations [10,11], the most recent national health strategic plans, national malaria control plans, health financing plans, Ministry of Health websites and correspondence with WHO offices and National Malaria Control Programme managers were used to define the boundaries of health districts in the 7 Sahelian countries and the nine states of northern Nigeria (Section A and B in S1 File).

Population density mapping techniques [12,13] were used to allocate population counts at 1 x 1 km resolutions across the study countries based on the most recent, highest resolution population census data and remotely sensed land-cover classification systems. This approach was extended to further classify populations into urban and rural based on population density, night time lights and other land cover classifications. The modelled population estimates were projected to 2015–2020 using national rural and urban growth rates estimated by the UN Population Division [14]. Data on sub-national population age structures was obtained from a variety of sources and used to derive adjustments of the population density by the age categories of 3 months to below 5 years; 5 years to below 10 years and ≥ 10 years (Section C in S1 File).

An approach developed by Cairns and colleagues [6] was adopted to define areas of acute seasonal transmission as those where 60% or more of the annual total rainfall occurred in three consecutive months. To replicate this approach, daily rainfall estimates from the Africa Rainfall Estimates version 2 (RFE 2.0) data from 2002–2009 [15] were resampled to 1 x 1 km spatial resolution and used to identify seasonal areas (Section D in S1 File). For each health district the proportion of the population that was located in areas where ≥60% of rainfall occurred within any 3 consecutive months was computed. Health districts were identified as seasonal if 80% of population lived in areas where > = 60% of rainfall occurred in 3 consecutive months.

Within health districts that were identified as seasonal, the median number of transmission months was extracted from a map obtained from the International Research Institute for Climate and Society website [16,17]. This map was developed from long-term rainfall and temperature data and their theoretical relationships with *P*. *falciparum* malaria transmission [17]. The map of number of transmission months was defined at spatial resolution of approximately 50 x 50 km and was resampled to 1 x 1 km to match the seasonality maps.

Recently published continuous maps predicted from community *Plasmodium falciparum* parasite rate (*Pf*PR) data standardised to the age range 2 to just below 10 years (*Pf*PR_2-10_) at 1 × 1 km spatial resolution for the year 2000 and 2010 was used to define receptive and current risks respectively [5]. The continuous *Pf*PR_2-10_ surfaces were used together with the population distribution surfaces for the same year at matching 1 × 1 km spatial resolutions to compute population adjusted *Pf*PR_2-10_ (PA*Pf*PR_2-10_) by health district for both 2000 and 2010. To compute PA*Pf*PR_2-10_, the mean proportion of the posterior *Pf*PR_2-10_ for a given year was multiplied with the pixel level population surface to estimate the numbers of people who were likely to be infected per pixel for that year, which was then summed for each health district. The estimated population that was infected was divided by the total population of the health districts for that year to generate the mean PA*Pf*PR_2-10_, (Section F in S1 File).

### SMC target health districts, population of children and antimalarial tablets

Health districts were identified as suitable for SMC if they were seasonal (>80% of population lived in areas where > = 60% of rainfall occurred in 3 consecutive months) and had 2000 PA*Pf*PR_2-10_ (receptive risk) of ≥5%. Within these SMC health districts, children 3 months to below 10 years were targeted in those where transmission was 5% to 10% PA*Pf*PR_2-10_ in 2010. In districts where transmission PA*Pf*PR_2-10_ in 2010 as >10%, SMC was targeted at children 3 months to below 5 years. Projected population of SMC targeted children by age class were extracted for each year from 2015 to 2020 for each health district. Using the year 2015 for illustration, the estimated number of SMC targeted children was multiplied by the median number of malaria transmission months per health district and the SP and AQ tablets required per child per month (Section G in S1 File).

## Results

**Fig 2:**
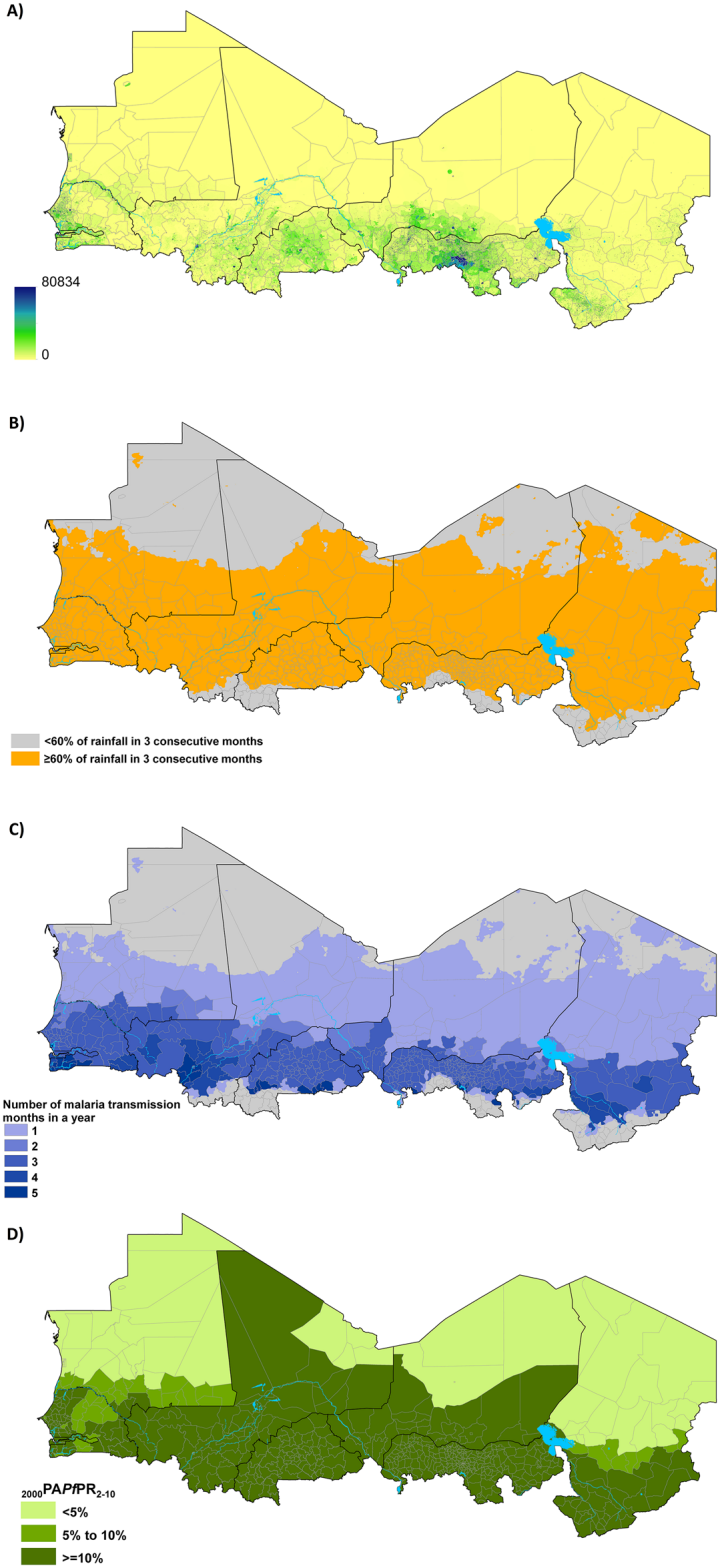
Map of Sahelian health districts (n = 597) showing A) the distribution of population in 2015 at 1 x 1 km spatial resolution [source: www.worldpop.org]; B) areas (orange) where 60% or more of the annual total rainfall occurs in any three consecutive months; C) the median number of malaria transmission months in an average year in seasonal health districts [source: Grover-Kopec et al 2006; D) population adjusted estimates of *P*. *falciparum* parasite rate standardized to the ages 2 to 10 years (PA*Pf*PR_2-10_) for the year 2000 [source: Noor et al 2014]. In Nigeria, health districts from the states of Bauchi, Borno, Jigawa, Kano, Katsina, Kebi, Sokoto, Yobe and Zamfara were included in the analysis.

Fig 2A shows population distribution map at 1 × 1 km spatial resolution, on which the boundaries of the updated health districts overlay, demonstrating an increasing density of population north to south at increasing distance from the Sahara Desert. Areas that have acute malaria seasonality, however, were concentrated in the middle Sahelian belt and excluded several of the extremely arid northern districts and the humid districts in the Equatorial south (Fig 2B). Within the seasonal areas the number of months of transmission increased southwards with most districts having a median 3 of months (Fig 2C). Estimated receptive *P*. *falciparum* transmission intensity, as measured by PA*Pf*PR_2-10_ in 2000 (Fig 2D), showed increasing rates north to south with almost all the districts in northern arid areas having infection rates of <5%.

**Fig 3:**
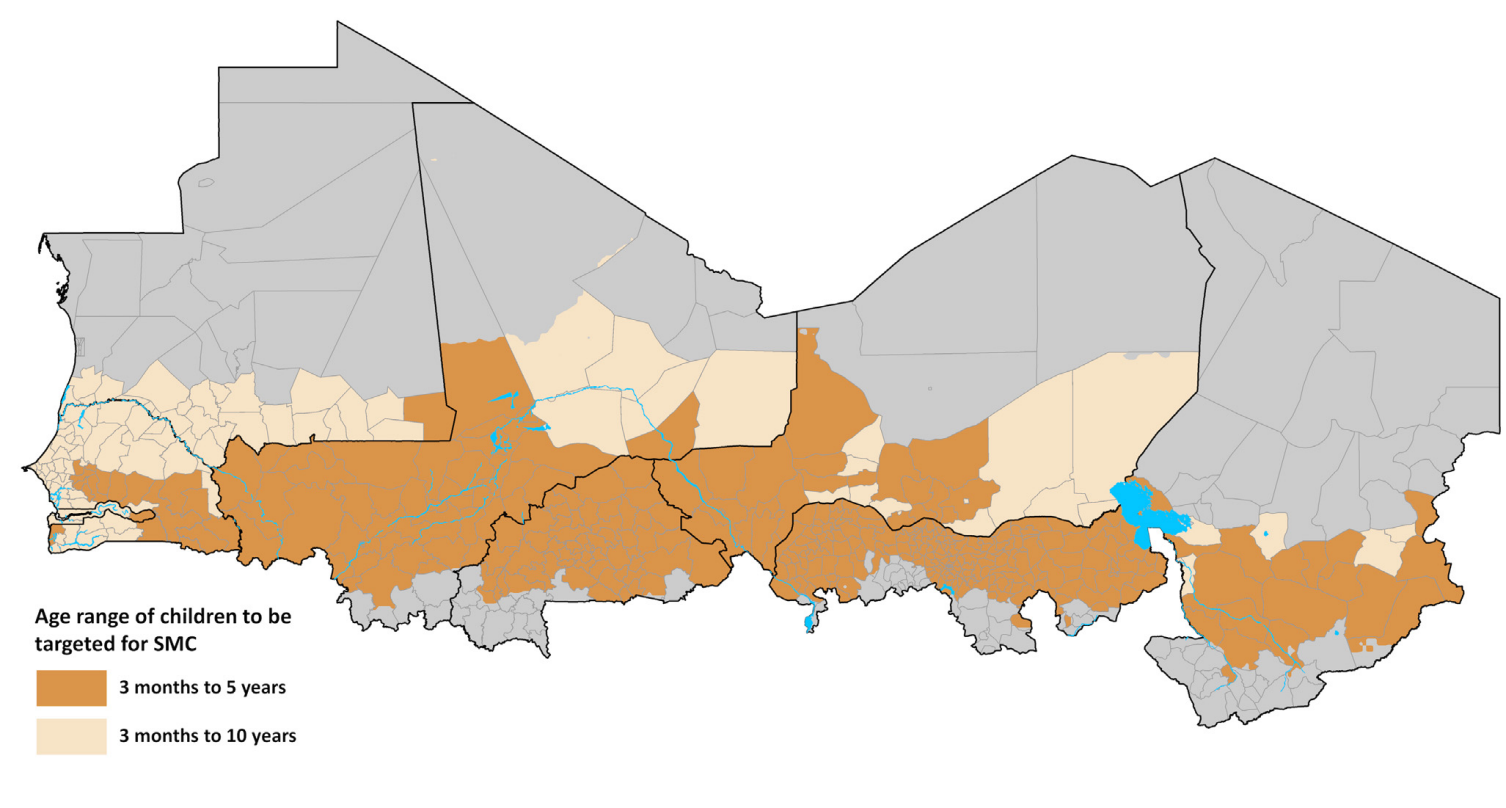
Map of the Sahel showing the health districts that are not suitable for SMC targeting (grey n = 119) and SMC suitable health districts (light to dark brown, n = 478) classified by age class of target children. The SMC suitable districts were those where PA*Pf*PR_2-10_ in 2000 was ≥5% and 80% of the population lived in areas where ≥60% or more of the annual total rainfall occurs in any three consecutive months. In SMC health districts where PA*Pf*PR_2-10_ in 2010 was ≥5% to 10% (n = 123) children 3 months to <10 years of age were targeted for SMC while those where it was >10% (n = 355) children 3 months to <5 years of age were targeted. *All inputs are either generated at or resampled to surfaces of 1 x 1 km spatial resolutions. **A**) Monthly Africa Rainfall Estimates version 2 (RFE 2.0) data from 2002–2009 at 10 × 10 km spatial resolution [NOAA 2013] were used to generate average long term monthly rainfall which are then used to define average seasonality (Section D in S1 File); **B**) Maps of total population are disaggregated by age structure (3 months to below 5 years; 5 years to below 10 years) using data from census and household surveys and by urban and rural using population density, night time lights and other land cover classifications (Section C in S1 File). Countries should use most recent census and survey data for population projections and age categorisations; **C**) For all countries except Niger and Mauritania *Pf*PR_2-10_ data from the period 1980–2012 were used to estimate endemicity from 2000 and 2010 (Section F in S1 File). **D**) A map based on the presumed relationship between *P*. *falciparum* transmission, temperature and rainfall to define the length of transmission seasons was downloaded as a grid surface from International Research Institute for Climate and Society website [IRI URL]. The map was at spatial resolution of approximately 50 x 50 km and was resampled to 1 x 1 km (Section E in S1 File); **E**) The approach by Cairns et al (2012) that identified acute malaria seasonality as areas where 60% or more of the annual total rainfall occurred in three consecutive months was used. This approach had a high sensitivity (95%) of areas where over 60% of malaria cases occurred in 4 consecutive months (Section D in S1 File,); **F**) Data from a variety of international and national sources were used to develop the most recent boundaries of health districts (Section B in S1 File). Due to population growth and changes in governance health districts change frequently and countries should continuously update these boundary changes. **G-J**) Health districts where ≥80% of population lived in areas of acute malaria seasonality and had 2000 PA*Pf*PR_2-10_ ≥ 5% were considered suitable for SMC (Section F in S1 File). This endemicity threshold allowed for the inclusion of areas where current risk is low but where receptive risk is still high. Population estimates by age class, urban and rural were extracted to each health district (S1 File); **K**) In districts where 2010 *Pf*PR_2-10_ was 5% to ≤10%, children aged 3 months to <10 years were targeted for SMC and 3 to months to <5 years in higher transmission districts (Section F in S1 File). Countries can update the contemporary description of risk using most recent survey data. A decision also needs to be made on whether or not to include urban areas. **L**) The median number of transmission months was extracted for each health district from the climate based map of length of transmission (Section E in S1 File) and was multiplied by the estimated number of SMC targeted children and the 1 SP and 3 AQ tablets per child per month (Section F in S1 File).

Out of the 597 Sahelian health districts in the eight countries, 543 (90.1%) had the defined receptive risk threshold suitable for SMC of ≥5% PA*Pf*PR_2-10_ in 2000 (Fig 2C and Table 1). A total of 514 health districts also met the seasonality criterion of 80% of the 2000 population living in areas where ≥60% of rainfall occurring in three consecutive months (Table 1). 478 (80.1%) health districts met both criteria of a 2000 endemicity of >5% PA*Pf*PR_2-10_ and 80% of the population lived in areas where ≥60% of rainfall occurring in three consecutive months and were therefore considered suitable for SMC (Table 1 and Fig 3). The health districts in the nine northern states of Nigeria (Bauchi, Borno, Jigawa, Kano, Katsina, Kebi, Sokoto, Yobe and Zamfara) accounted for about 38% of all SMC suitable districts (Table 1). In The Gambia and Senegal all the health districts were identified as suitable for SMC. An estimated 85% of the population in the Sahel or 119.8 million in 2015, increasing to 139.1 million by 2020, were in the SMC suitable health districts (Table 1).

**Table 1:** A summary of the health districts and population in the countries of the Nouakchott Initiative for targeting of seasonal malaria chemoprevention (SMC) in the Sahel from 2015–2020.

	Burkina Faso	Chad	Gambia	Mali	Mauritania	Niger	Nigeria	Senegal	Total
**Total number of districts**	70	62	7	60	53	42	227	76	**597**
**Number of districts where PA*Pf*PR**_**2-10**_ **was greater 5%**	70	44	7	56	26	38	227	75	**543**
**Number of districts where >80% of population lived in seasonal areas**	57	39	7	53	43	41	198	76	**514**
**Number of districts suitable for SMC**	57	25	7	52	26	38	198	75	**478**
**Number of SMC districts children 3 months to 10 years should be targeted (5% to ≤10% PA*Pf*PR**_**2-10**_ **in 2010)**	0	11	7	5	26	12	0	62	**123**
**Total population (millions) by year in SMC targeted districts**									
**2015**	15.7	6.3	2.0	14.8	2.4	18.6	45.3	14.7	**119.8**
**2016**	16.2	6.5	2.0	15.3	2.4	19.4	46.5	15.1	**123.4**
**2017**	16.7	6.7	2.1	15.8	2.5	20.1	47.7	15.5	**127.1**
**2018**	17.2	6.9	2.2	16.3	2.5	20.9	49.0	16.0	**131.0**
**2019**	17.7	7.1	2.2	16.8	2.6	21.8	50.4	16.5	**135.1**
**2020**	18.2	7.3	2.3	17.3	2.6	22.6	51.8	16.9	**139.0**
**Total**	**101.7**	**40.8**	**12.8**	**96.4**	**14.9**	**123.4**	**290.6**	**94.7**	**775.3**

If the WHO recommendation of targeting only children 3 months to below five years was applied, a total of 158 million children, including 121.5 million in rural areas, will need SMC to achieve universal coverage in all targeted health districts in the six years from 2015–2020 (Table 2). Approximately 40% of these children were from Nigeria regardless of whether all or only rural districts were targeted. Burkina Faso, Mali, Niger and Senegal were the other countries that contributed the largest target population and together with Nigeria accounted for over 90% of all targeted children in the SMC districts. The countries where the exclusion of the urban areas resulted in the largest absolute reductions in population were Nigeria (13.5 million), Senegal (5.7 million), Mali (5.4 million) and Burkina Faso (5.3 million).

**Table 2:** Estimated target population (millions) of children in SMC health districts in in the countries of the Nouakchott Initiative from 2015–2020 by age range and residence.

Target population	Age class of target children	Year	Burkina Faso	Chad	Gambia	Mali	Mauritania	Niger	Nigeria	Senegal	Total
	**3 months to below 5 years in all districts**	2015	2.6	1.1	0.3	2.7	0.4	3.6	9.2	2.3	22.2
		2016	2.8	1.2	0.4	2.9	0.4	4.1	10	2.5	24.2
		2017	2.9	1.3	0.4	3.1	0.4	4.4	10.5	2.6	25.6
		2018	3	1.3	0.4	3.3	0.4	4.7	11.1	2.8	27.1
		2019	3.2	1.4	0.4	3.5	0.4	5.1	11.7	2.9	28.7
		2020	3.3	1.5	0.5	3.7	0.4	5.5	12.3	3.1	30.4
**All**	**Total**		**17.7**	**7.7**	**2.4**	**19.2**	**2.4**	**27.4**	**64.8**	**16.3**	**158**
	**3 months to below 10 years in SMC districts with PA*Pf*PR**_**2-10**_ **5% to ≤10% and 3 months to below 5 years in districts >10% PA*Pf*PR**_**2-10**_	2015	2.6	1.4	0.6	2.8	0.7	4.4	9.2	4.1	25.7
		2016	2.8	1.5	0.6	3	0.7	4.8	10	4.2	27.5
		2017	2.9	1.5	0.6	3.2	0.7	5	10.5	4.3	28.8
		2018	3	1.6	0.6	3.4	0.7	5.3	11.1	4.3	30.1
		2019	3.2	1.6	0.7	3.6	0.7	5.6	11.7	4.5	31.6
		2020	3.3	1.7	0.7	3.8	0.8	6.1	12.3	4.7	33.5
	**Total**		**17.7**	**9.4**	**3.8**	**19.9**	**4.3**	**31.2**	**64.8**	**26.1**	**177.1**
	**3 months to below 5 years in all districts**	2015	1.9	0.8	0.2	2	0.4	3.2	7.5	1.5	17.4
		2016	2	0.9	0.2	2.1	0.4	3.5	8	1.7	18.8
		2017	2	1	0.2	2.3	0.4	3.8	8.4	1.7	19.7
		2018	2.1	1	0.2	2.4	0.4	4.1	8.8	1.8	20.8
		2019	2.2	1.1	0.3	2.5	0.4	4.4	9.2	1.9	21.8
		2020	2.2	1.1	0.3	2.6	0.4	4.7	9.6	2	23
**Rural**	**Total**		**12.4**	**5.9**	**1.4**	**13.9**	**2.4**	**23.7**	**51.3**	**10.6**	**121.5**
	**3 months to below 10 years in SMC districts with PA*Pf*PR**_**2-10**_ **5% to ≤10% and 3 months to below 5 years in districts >10% PA*Pf*PR**_**2-10**_	2015	1.9	1	0.3	2.1	0.7	3.8	7.5	2.7	19.8
		2016	2	1	0.3	2.2	0.7	4.1	8	2.7	21.1
		2017	2	1.1	0.4	2.3	0.7	4.3	8.4	2.8	22
		2018	2.1	1.1	0.4	2.5	0.7	4.6	8.8	2.8	22.9
		2019	2.2	1.2	0.4	2.6	0.7	4.8	9.2	2.9	23.9
		2020	2.2	1.2	0.4	2.7	0.8	5.2	9.6	3	25.1
	**Total**		**12.4**	**6.6**	**2.2**	**14.3**	**4.3**	**26.8**	**51.3**	**16.9**	**134.7**

If the age of children to be targeted with SMC was expanded to children 3 months to below 10 years in 123 health districts where PA*Pf*PR_2-10_ was 5% to ≤10% in 2010 a total of 177.1 children, including 134.7 million in rural areas, would require SMC between 2015–2020 (Table 2). The countries most affected by the expansion of the age of children were Senegal and Niger where additional 9.8 million and 3.7 million children required SMC. There were no health districts in Burkina Faso that met the criterion for including older children.

The amount of tablets required for 2015 was computed from the population size of targeted children (Table 2), the number of SP and AQ tablets per transmission month based on WHO recommendations and the median number of malaria transmission months per year (Fig 1C). It is estimated that if children 3 months to below 5 years of age across all SMC districts were targeted, 64 million SP and 192 million AQ tablets would be required in 2015 (Table 3). If, however, children 3m to <10 years were additionally covered in health districts where PA*Pf*PR_2-10_ was 5% to ≤10% in 2010, 72 million SP and 217 million AQ tablets will be required. Focusing on rural children only reduced the amount of tablets required from 49 million and 55 million SP tablets and 148 and 164 million AQ tablets depending on the ages of children included.

**Table 3:** Estimated numbers of SP and AQ tablets required in SMC health districts in in the countries of the Nouakchott Initiative in 2015. To compute the total tablets, the estimated population of targeted children was multiplied by the number of tablets per child for SP and AQ separately and the number of months of transmission in a health district. The WHO guidelines [WHO 2013] recommend that a child is given one tablet of SP and 3 tablets of AQ during each transmission month. In 24 health districts where the estimated median months of transmission was >4, we have assumed a 4-month transmission season to adhere to the WHO recommendation that SMC should last no more than 4 months.

**Target population**	**Age class of target children**	**Drug**	**Burkina Faso**	**Chad**	**Gambia**	**Mali**	**Mauritania**	**Niger**	**Nigeria**	**Senegal**	**Total**
**All**	**3 months to below 5 years in all districts**	**SP**	7.8	3.5	1.2	8.4	0.7	7.5	27.9	7	64
		**AQ**	23.5	10.5	3.6	25.1	2.2	22.4	83.8	20.9	192.1
	**3 months to below 10 years in SMC districts with PAPfPR2-10 5% to ≤10% and 3 months to below 5 years in districts >10% PAPfPR2-10**	**SP**	7.8	4.4	2.1	8.5	1.5	8.9	27.9	11.4	72.4
		**AQ**	23.5	13.1	6.4	25.5	4.4	26.6	83.8	34.1	217.3
**Rural**	**3 months to below 5 years in all districts**	**SP**	5.7	2.7	0.7	6	0.7	6.3	22.7	4.6	49.4
		**AQ**	17	8	2	17.9	2.2	19	68	13.9	148.2
	**3 months to below 10 years in SMC districts with PAPfPR2-10 5% to ≤10% and 3 months to below 5 years in districts >10% PAPfPR2-10**	**SP**	5.7	3.1	1.1	6.1	1.5	7.5	22.7	7.3	54.8
		**AQ**	17	9.2	3.4	18.2	4.4	22.4	68	21.8	164.4
**Target population**	**Age class of target children**	**Drug**	**Burkina Faso**	**Chad**	**Gambia**	**Mali**	**Mauritania**	**Niger**	**Nigeria**	**Senegal**	**Total**
**All**	**3 months to below 5 years in all districts**	SP	7.8	3.5	1.2	8.4	0.7	7.5	27.9	7	64
		AQ	23.5	10.5	3.6	25.1	2.2	22.4	83.8	20.9	192.1
	**3 months to below 10 years in SMC districts with PA*Pf*PR**_**2-10**_ **5% to ≤10% and 3 months to below 5 years in districts >10% PA*Pf*PR**_**2-10**_	SP	7.8	4.4	2.1	8.5	1.5	8.9	27.9	11.4	72.4
		AQ	23.5	13.1	6.4	25.5	4.4	26.6	83.8	34.1	217.3
**Rural**	**3 months to below 5 years in all districts**	SP	5.7	2.7	0.7	6	0.7	6.3	22.7	4.6	49.4
		AQ	17	8	2	17.9	2.2	19	68	13.9	148.2
	**3 months to below 10 years in SMC districts with PA*Pf*PR**_**2-10**_ **5% to ≤10% and 3 months to below 5 years in districts >10% PA*Pf*PR**_**2-10**_	SP	5.7	3.1	1.1	6.1	1.5	7.5	22.7	7.3	54.8
		AQ	17	9.2	3.4	18.2	4.4	22.4	68	21.8	164.4

## Discussion

National Malaria Control Programmes in the Sahel, that form part of the Nouakchott Initiative, have previously used a wide range of risk maps to coordinate their activities (S1 File). These maps, which are expert-opinion, based on climate suitability or aggregated representations of parasite prevalence from recent household surveys, do not provide a single standard metric across countries for sub-national SMC planning. They are also not presented in ways that are helpful to allocate resources within health decision-making units. The sub-national SMC targeting approach we have developed in this study not only recognizes the need to support decentralized health decision-making but provides a common decision making platform across the countries of the Nouakchott Initiative. It also potentially provides a complementary frontend for the SMC implementation toolkit developed by the WHO Global Malaria Programme, in collaboration with the Medicines for Malaria Venture (MMV) [18] that focuses on planning, training and communication to guide the process of actual delivery of the intervention to communities and subsequent monitoring and evaluation.

Our analysis shows that over 85% of all the population of the Nouakchott Initiative region live in areas that are suitable for SMC, and the nine states of Nigeria alone contribute 40% of this population. The WHO currently recommends that only children 3 months and below 5 years of age are targeted with SMC in the Sahel and sub-Sahel [3] resulting in 158 million children that need to be reached with the intervention over the period 2015–2020. Approximately 34 million of these children are from urban areas. However, there are no clear guidelines on whether urban populations in SMC suitable areas should be targeted for malaria chemoprevention. Overall malaria infection rates are lower in urban compared to neighbouring rural areas of Africa [19, 20] but may have areas of high receptivity that are at risk of upsurge in transmission due seasonal human population movements [21] suggesting a potential benefit from SMC. There are also many other features of urban life not captured by exposure risk alone, including accessibility to curative services and household economies but the evidence on the efficacy of SMC in urban areas remains limited.

In addition to seasonality, an accurate understanding of malaria endemicity is key to identifying areas suitable for SMC. In this study we argue that the use of contemporary measures of malaria risk to identify SMC suitable areas [6] would exclude districts where malaria incidence has reduced to low levels [5] but still have substantial transmission potential. For example, the map for Senegal that is currently used for planning [Fig A in S1 File] is one of regional summary of parasite prevalence from the 2011 malaria indicator survey highlighting the current low prevalence and suggests that large parts of Senegal, on the basis of transmission intensity and corresponding disease incidence, are not suited to SMC. The use of contemporary malaria endemicity maps also assumes that ITN coverage [22] or resistance to pyrethroids in the Sahel [23,24] will remain at current levels. These are both high-risk scenarios given the current financial constraints to overseas development assistance and domestic health funding [22] and the presence of pyrethroid resistance in the sub-region. Instead, we have used estimates of *P*. *falciparum* transmission in 2000, prior to the large scale up of malaria control in Africa and a reasoned estimate of receptive risks, to define endemicity thresholds for SMC suitability.

We propose an alternative role for contemporary maps of malaria endemicity where, instead of using them to define endemicity thresholds for SMC suitability, we apply them to determine where to expand the upper age limit of children to be targeted. In areas where transmission has declined, such as in various parts of the Sahel and sub-Sahel [5], in part due to malaria control interventions including SMC, the age pattern of *P*. *falciparum* infections and disease are likely to decline with more older children becoming ill [7, 8, 25]. Consequently, the impact of SMC on the burden of malaria may be greater if children above the age of five years are targeted. There is no clear evidence on the upper age limit of children for inclusion or the exact levels of transmission that determine when such a switch should be made. However, the literature suggests a shift in the age pattern of infection and disease occurs under hypoendemic transmission conditions [7, 8], corresponding to PA*Pf*PR_2-10_ of ≤10%. In this study, we estimate that if children up to <10 years of age were included in heath districts where PA*Pf*PR_2-10_ of was 5% to ≤10%, an additional 19 million children overall, including 15 million children in rural areas, would require SMC in the period 2015–2020. Approximately 10 million of these children would be from Senegal, the only Nouakchott Initiative country that has already decided to expand the age class of SMC targeted children to include those under 10 years of age. However, as local data on the age distribution of malaria become available, and evidence on the benefits of increasing the upper age limits accumulates, countries may consider increasing the age of children to be targeted for SMC to beyond 10 years.

Our analysis indicates that Nigeria (northern), Mali and Burkina Faso account for over 60% of all children living in areas suitable for SMC. From a Nouakchott Initiative regional perspective a bigger impact may be achieved by prioritising these countries. However, the potential impact of SMC is not determined by the size of the population at risk alone but also the strength of the health system tasked to deliver the intervention, the availability of domestic resources in addition to external funding to sustain coverage and other factors such as conflict, general good governance and conflict. In addition, where regional initiatives exist, prioritising one country over another, without the requisite political goodwill, may lead to tension. Decisions on prioritising high population at risk and potentially high return countries must therefore be done on the basis of regional agreements and the existing operational environment to ensure maximum impact. Overall, the primary objective should be that resources are made available to all those countries that are likely to benefit from SMC.

The reliability of the various inputs into the spatial decision support framework drive the overall accuracy of the definition of the SMC target health districts and the quantification of the size of the target population of children and the required tablets. Wide variations exist in the number of transmission months within the SMC suitable areas of Sahel and sub-Sahel [17], key factor to quantifying the required tablets and other operational inputs. In our analysis we incorporate this information into the decision process and show that up to 289 million combined tablets of AS and AQ would be required in 2015 alone to achieve universal coverage across the region. Despite, their obvious epidemiological and operational importance [1, 26] definitions of malaria seasonality, start and length of seasons still remain poorly described. Various theoretical relationships between transmission intensity and climate indicators, specifically temperature, rainfall and humidity, have been used to define malaria seasonality, its beginning and its length [6, 17, 27]. In our current analysis, several districts in the southern margins of the Sahel are classified to have five or six median number of transmission months. This could be as a result of the uncertainties in the maps of transmission months we used [17] or that of the seasonality surface (Fig 2A), whose bounds may be covering areas of high transmission where seasonality is less acute with longer transmission period. For these districts, we suggest that countries use the WHO recommendation of up to 4 months of SMC intervention [3]. Further work using larger empirical malaria and higher resolution climate data are, however, required to improve the precision of these seasonality models. Studies in the agriculture sector on forecasting of rainfall seasons to inform the crop-growing periods in rainfed crop production systems in the Sahel [28, 29, 30] could provide useful lessons for SMC.

The model-based geostatistical methods use to predict *Pf*PR_2-10_ indicate reasonable accuracy based on the prediction to a carefully selected holdout dataset [5]. However, our estimates of contemporary risk are to 2010 and countries need to update these maps as new survey data become available. This will help the continuous process of deciding when and where to update the upper age limit of children to be targeted for SMC. The population distribution maps, which are used to compute both the PA*Pf*PR_2-10_ and the size of SMC population, are also based on modelled redistribution of census data, the accuracy of which is largely determined by the spatial resolution and the currency of this data [12]. All the countries in this study have had a census since 2009, except for Burkina Faso and Nigeria where the last censuses were done in 2006 [Table B in S1 File]. Despite the recent censuses, data used to develop the high-resolution population maps were only available at the second administrative unit, the equivalent of a district, for Chad, The Gambia and Mauritania.

Finally, we have considered *P*. *falciparum*, by far the most pathogenic of the four malaria parasites found in Africa. However, the Sahel sub-region does support *P*. *vivax* transmission and significant numbers of clinical cases of *P*. *vivax* have been documented in Mauritania, and increasingly in and around Nouakchott [31, 32]; Mali [33, 34], Burkina Faso [35] and Niger [36]. Further work is required to define the true spatial extent of *P*. *vivax* in the Sahel and investigate the clinical significance of this parasite and its responsiveness to SMC using SP+AQ.

## Conclusions

Our spatial decision support framework allows for a common platform for decision making for SMC for the countries of the Nouakchott Initiative. From the estimated numbers of children to be targeted with SMC for the period 2015–2020 and tablets required for 2015 alone, the region clearly needs to invest substantial resources to reach universal coverage of SMC in areas that are likely to benefit the most. Where the financial and opportunity costs of various delivery mechanisms are known [37], our empirical estimates of SMC target population could provide the denominator to support comprehensive costing of the SMC scale up across the Sahel and is easily compatible with existing implementation toolkit from the WHO [16]. Several challenges remain, however, including availability of sufficient prevalence data in Niger and Mauritania, improving the measures of seasonality, the start and length of the transmission season, the place of urban population in malaria chemoprevention and the understanding the true prevalence of *P*. *vivax* and the role of SMC to reduce its burden in the Sahel. In addition, there are likely to be other sub Saharan African countries outside of the Sahel that meet both the disease burden and seasonality criteria for SMC suitability [6] but where resistance to both SP and AQ are high [3]. Experimental evaluations of different therapeutic agents are required to expand the benefits of SMC to these countries.

## Supporting Information

S1 File Supplementary Information for Sub-national targeting of seasonal malaria chemoprevention in the Sahelian countries of the Nouakchott Initiative. (DOCX)
