# Correction: Sub-National Targeting of Seasonal Malaria Chemoprevention in the Sahelian Countries of the Nouakchott Initiative

**DOI:** 10.1371/journal.pone.0140414

**Published:** 2015-10-07

**Authors:** Abdisalan Mohamed Noor, Eliud Kibuchi, Bernard Mitto, Drissa Coulibaly, Ogobara K. Doumbo, Robert W. Snow

Fig 1 and Fig 3 are incorrect. Both figure legends are incorrect. The authors have provided a corrected version here.

**Fig 1:**
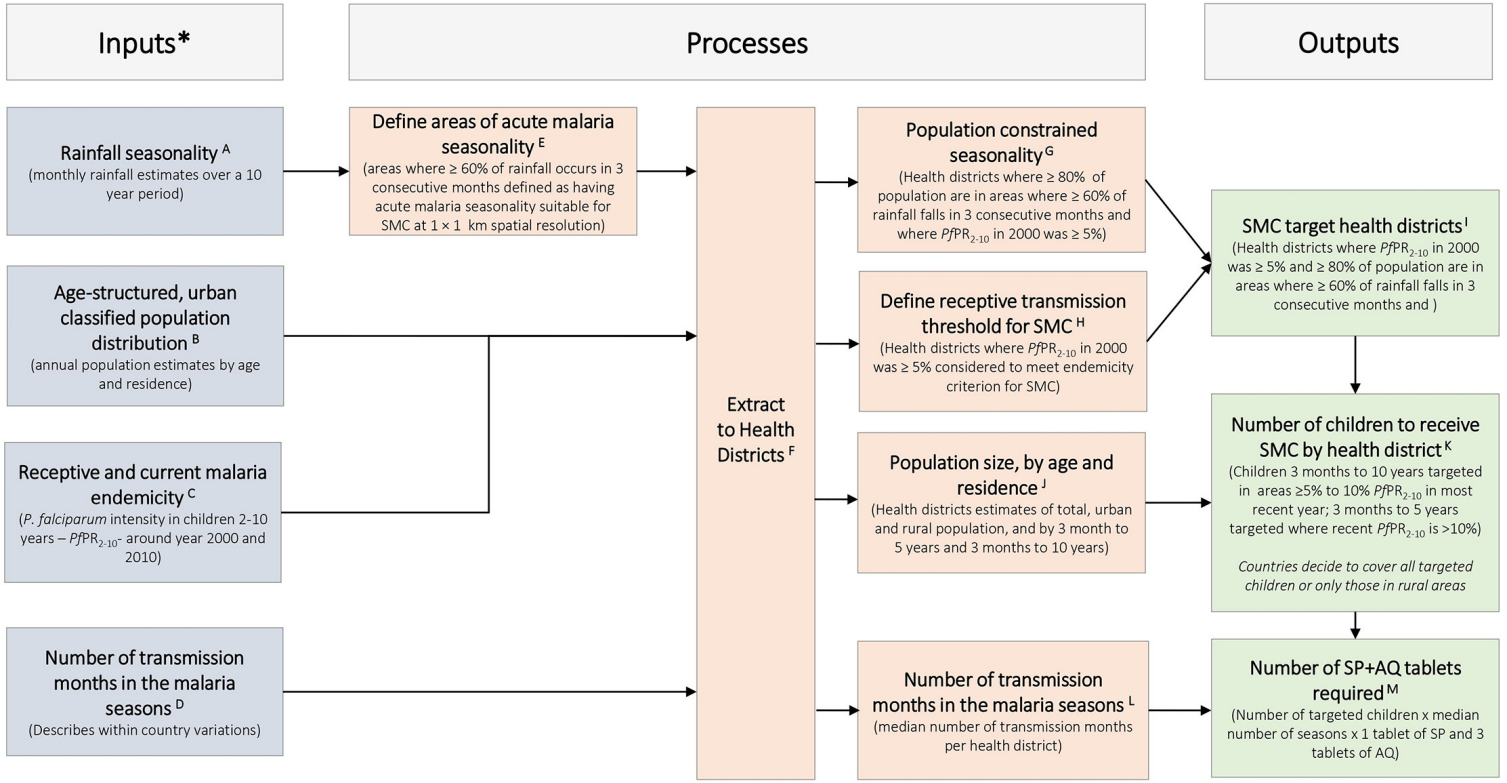
A spatial decision support framework for identifying areas suitable for seasonal chemoprevention and quantifying the size of the population of target children and the amount of the required antimalarial tablets. The SMC suitable districts were those where PAPfPR2-10 in 2000 was 5% and 80% of the population lived in areas where 60% or more of the annual total rainfall occurs in any three consecutive months. In SMC health districts where PAPfPR2- 10 in 2010 was 5% to 10% (n = 123) children 3 months to 10% (n = 355) children 3 months to <5 years of age were targeted. *All inputs are either generated at or resampled to surfaces of 1 x 1 km spatial resolutions. **A**) Monthly Africa Rainfall Estimates version 2 (RFE 2.0) data from 2002–2009 at 10 × 10 km spatial resolution [NOAA 2013] were used to generate average long term monthly rainfall which are then used to define average seasonality (Section D in S1 File); **B**) Maps of total population are disaggregated by age structure (3 months to below 5 years; 5 years to below 10 years) using data from census and household surveys and by urban and rural using population density, night time lights and other land cover classifications (Section C in S1 File). Countries should use most recent census and survey data for population projections and age categorisations; **C**) For all countries except Niger and Mauritania *Pf*PR_2-10_ data from the period 1980–2012 were used to estimate endemicity from 2000 and 2010 (Section F inS1 File). **D**) A map based on the presumed relationship between *P*. *falciparum*transmission, temperature and rainfall to define the length of transmission seasons was downloaded as a grid surface from International Research Institute for Climate and Society website [IRI URL]. The map was at spatial resolution of approximately 50 x 50 km and was resampled to 1 x 1 km (Section E in S1 File);**E**) The approach by Cairns et al (2012) that identified acute malaria seasonality as areas where 60% or more of the annual total rainfall occurred in three consecutive months was used. This approach had a high sensitivity (95%) of areas where over 60% of malaria cases occurred in 4 consecutive months (Section D in S1 File,); **F**) Data from a variety of international and national sources were used to develop the most recent boundaries of health districts (Section B in S1 File). Due to population growth and changes in governance health districts change frequently and countries should continuously update these boundary changes. **G-J**) Health districts where ≥80% of population lived in areas of acute malaria seasonality and had 2000 PA*Pf*PR_2-10_ ≥ 5% were considered suitable for SMC (Section F in S1 File). This endemicity threshold allowed for the inclusion of areas where current risk is low but where receptive risk is still high. Population estimates by age class, urban and rural were extracted to each health district (S1 File); **K**) In districts where 2010 *Pf*PR_2-10_ was 5% to ≤10%, children aged 3 months to <10 years were targeted for SMC and 3 to months to <5 years in higher transmission districts (Section F in S1 File). Countries can update the contemporary description of risk using most recent survey data. A decision also needs to be made on whether or not to include urban areas. **L**) The median number of transmission months was extracted for each health district from the climate based map of length of transmission (Section E in S1 File) and was multiplied by the estimated number of SMC targeted children and the 1 SP and 3 AQ tablets per child per month (Section F in S1 File). For additional details of the definition of inputs, processes and outputs see the S1 File.

**Fig 3:**
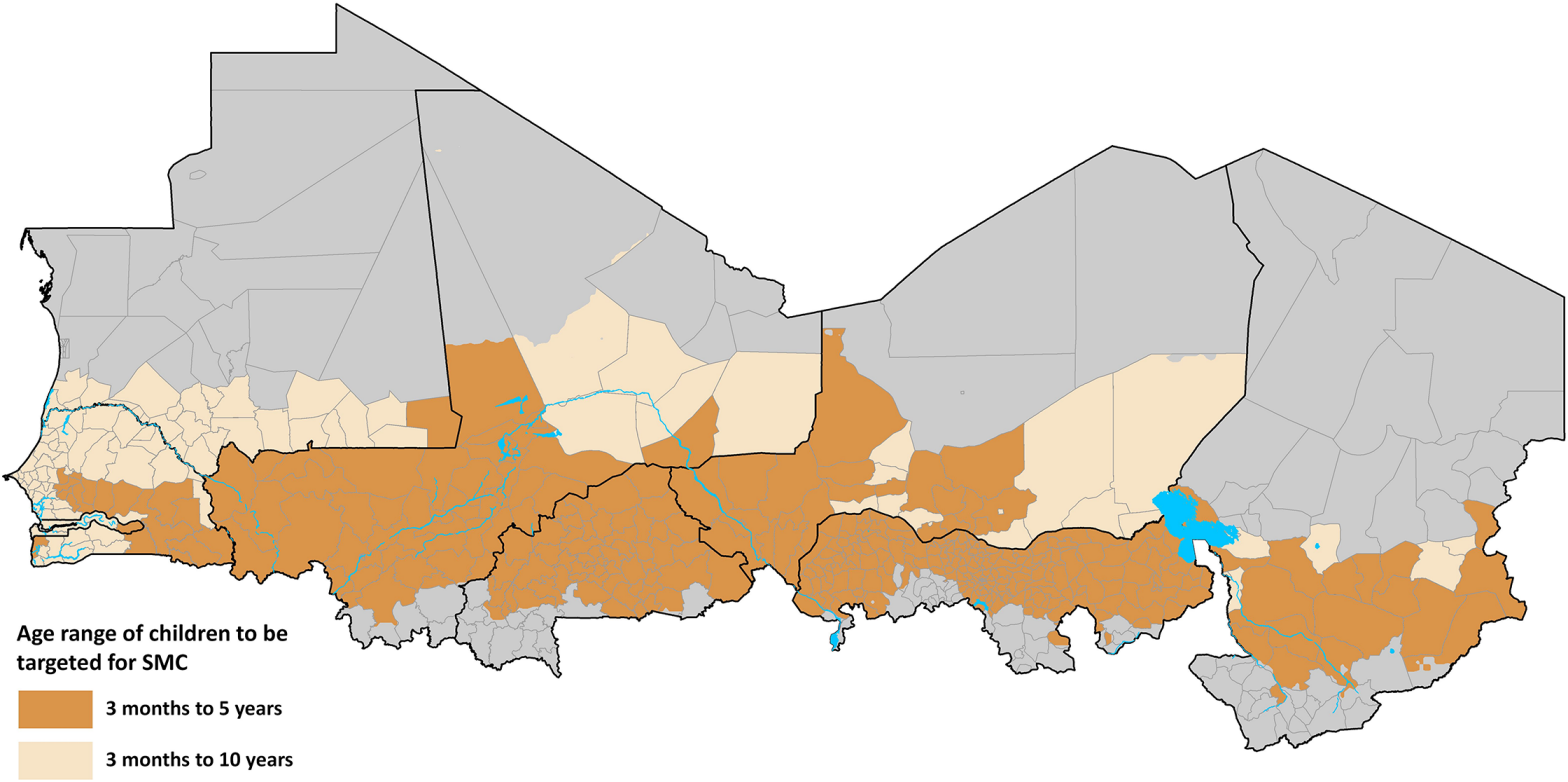
Map of the Sahel showing the health districts that are not suitable for SMC targeting (grey n = 119) and SMC suitable health districts (light to dark brown, n = 478) classified by age class of target children.
