# Supplementary material for: Sub-National Targeting of Seasonal Malaria Chemoprevention in the Sahelian Countries of the Nouakchott Initiative
Source: PLoS One. 2015 Aug 31;10(8):e0136919. doi: 10.1371/journal.pone.0136919 (PMC4554730; doi:10.1371/journal.pone.0136919)
Supplement: S1 File — (DOCX) [file pone.0136919.s001.docx]

**Supplementary 1 File: Sub-national targeting of seasonal malaria chemoprevention in the Sahelian countries of the Nouakchott Initiative**

**Abdisalan M Noor^1,2*^, Eliud Kibuchi^1^, Bernard Mitto^1^, Drissa Coulibaly^3^, Ogobara K Doumbo^3^, Robert W Snow^1,2^**

^1^INFORM (Information for Malaria – www.inform-malaria.org), Spatial Health Metrics Group, Kenya Medical Research Institute/Wellcome Trust Research Programme, Box 00100-43640, Nairobi, Kenya.

^2^Centre for Tropical Medicine and Global Health, Nuffield Department of Clinical Medicine, University of Oxford, CCVTM, Oxford OX3 7LJ, UK.

^3^Malaria Research and Training Center, University of Sciences, Techniques and Technologies, Bamako, Mali B.P. E.1805, Bamako, Mali.

*Author for Correspondence: [anoor@kemri-wellcome.org](mailto:anoor@kemri-wellcome.org)

**Section A: Maps of malaria risk currently used in the Sahel**

The expert-opinion, climate-based or crude parasite prevalence maps shown in Fig. A and currently used by national governments to design and target malaria interventions do have a broad congruence with empirically modelled predictions [1]. These maps do not provide a single reproducible standard metric across countries for sub-regional planning nor can they be used to allocate resources within countries as part of a decentralized health system because information is not disaggregated to the health districts.

Fig. A Selection of maps used by countries within the Nouakchott Initiative to define sub-national risk

| 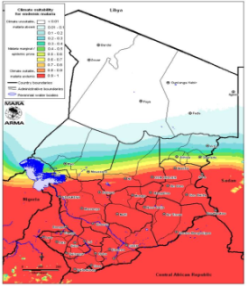 | **Chad**: Based on climate suitability or the likelihood of stable transmission using a rules-based, fuzzy logic approach based on long-term rainfall and temperature data [Craig et al., 1999]. The models assigns fuzzy values between 0 (unsuitable) and 1 (suitable) |
| --- | --- |
| 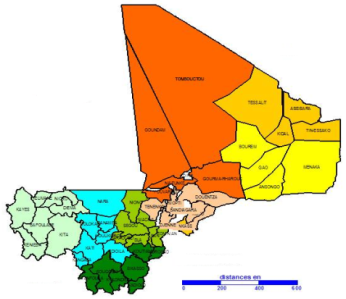 | **Mali**: Based on climate and eco-zonation regions, rivers and dams and uni- versus bimodal rainfall. |
| 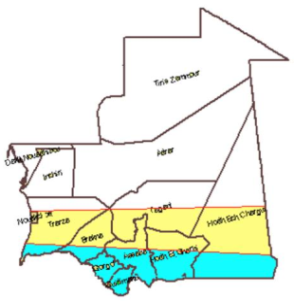 | **Mauritania**: based on eco-zonation: Sahelian Zone: located along the Senegal River Basin where malaria transmission is seasonal long (4-6months); Sahel-Saharan region: located in the south-east of the country between the Senegal River Valley Nouakchott and Nema-line is characterized by an annual rainfall of 100-300 mm and malaria transmission is seasonal short (2-4months): Sahara: a huge area north of Nouakchott and Nema online and malaria transmission is sporadic |
| 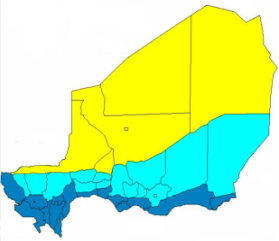 | **Niger**: Three epidemiological areas: North hypo endemic area (Saharan) with low transmission; meso intermediate zone (Sahel) with a short seasonal transmission (2 months); and hyper-endemic South (Sudanese zone) with a long regular seasonal transmission (> 6 months). |
| 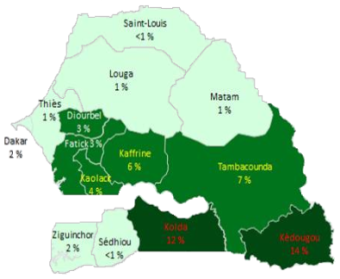 | **Senegal**: Regions based on 2011 parasite prevalence from Malaria Indicator Survey; highlighting the current low prevalence and ironically suggesting large parts of Senegal not suited to SMC |

**Section B: Reconciling information to health decision making units**

For the purpose of planning health service provision sub-nationally, most sub-Saharan African countries have undergone a process of decentralization. These health-decision making units are critical for targeted planning of federal or national resources. Federal decisions to target health resources below national recommendations are made at sub-national administrative levels created to support decentralized health care systems. These decision-making units are the lowest level of resource allocation, below this level very focal control demands a level of sophistication and cartography only reasonable to expect of malaria elimination strategies [2].

**Fig. B** 597 health administration districts used for planning service delivery in eight Sahelian countries. For Nigeria only nine northern states have been targeted for SMC: Bauchi, Borno, Jigawa, Kano, Katsina, Kebbi, Sokoto, Yobe and Zamfara.

**
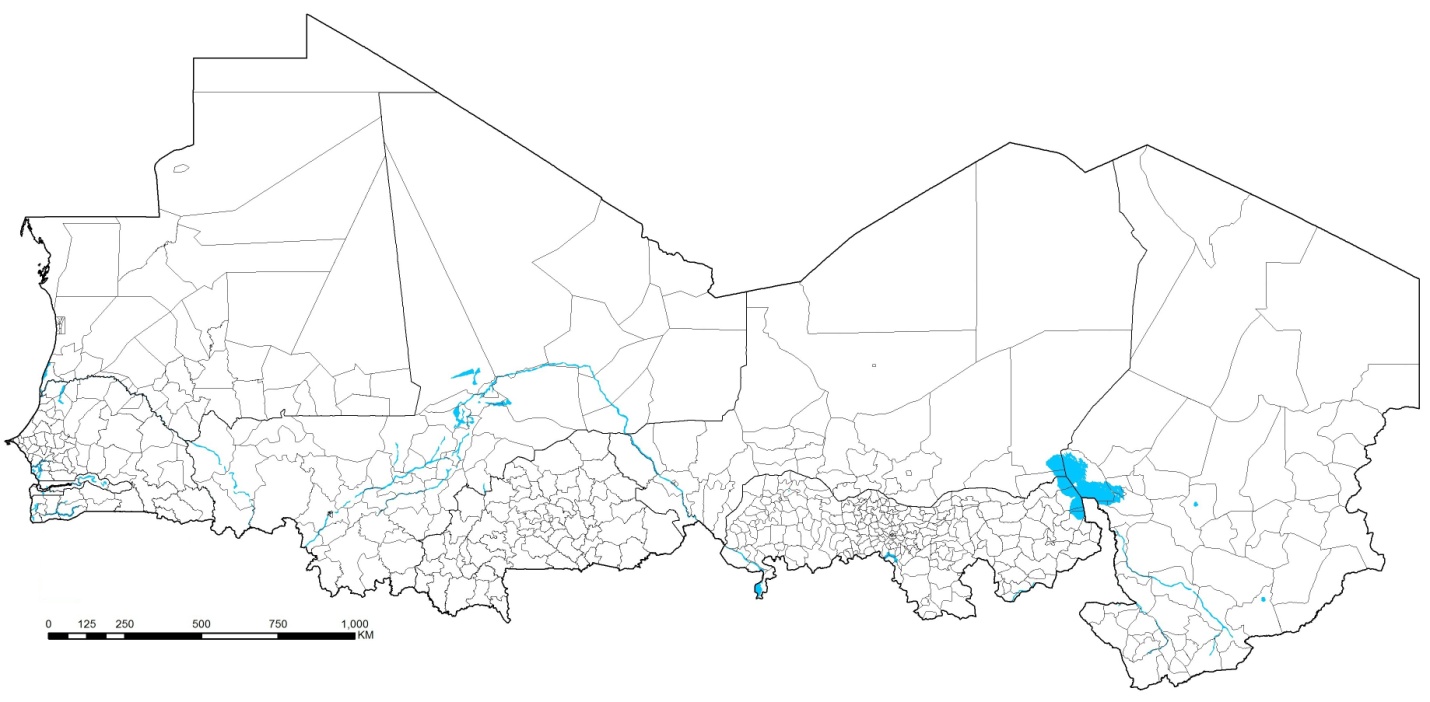
**

Health districts in the Sahel do not always conform to second-level administrative units used for national censuses, political governance or electoral constituencies or publicly available UN approved second-Level administrative boundary demarcations [3,4]. To update the health district maps we have identified the most recent national health strategic plans, national malaria control plans, health financing plans, ministry of health websites and correspondence with WHO offices and National Malaria Control Programme (NMCP) managers for each of the eight Sahelian countries. Where there were discrepancies with UN approved national and first-level boundaries we have digitised revised boundary levels using ArcGIS (ArcMap 10.1, ESRI Systems, Redland, CA, USA). The numbers of health decision-making units per country are shown Fig. B and described in Table A with the accompanying sources.

**Table A** Levels of health administration in Sahelian countries and data/ information sources used

| **Country** | **Admin level used**  **[Number of units]** | **Sources consulted to derive Health admin boundaries** |
| --- | --- | --- |
| Burkina Faso | District Sanitaire  [**69**] | Ministère de la Sante (2011). Plan stratégique de lutte contre le paludisme 2011-2015  http://www.sante.gov.bf/ |
| Chad | District Sanitaire  [**62**] | UNOCHA (2012). Humanitarian Response COD-FOD registry. Accessed 07 November, 2013 from https://cod.humanitarianresponse.info/search/field_country_region/102  Ministry of Public Health (2011). Emergency plan to interrupt the circulation of wild poliovirus in Chad: June - November 2011  http://www.sante-tchad.org/ |
| Gambia | Health Regions  [**7**] | Ministry of Health and Social Welfare (2012). National Health Policy 2012-2020. Accessed 13 November 2013 from http://www.nationalplanningcycles.org/sites/default/files/country_docs/Gambia/gambia_national_health_policy_2012-2020_mohsw.pdf  United Nations System in The Gambia (2011). United Nations Development Assistance Framework (UNDAF) 2012 – 2016 The Gambia. Accessed 13 November 2013 from http://www.gm.undp.org/UNDAF%20Final%203Oct.2011.pdf  http://www.statehouse.gm/health.html  Personal communication Dr Tom Sukwa, WR-WHO, The Gambia |
| Mali | District Sanitaire  [**60**] | Ministère de la Sante (2006). Plan Stratégique de Lutte Contre le Paludisme 2007-2011.  President’s Malaria Initiative (2013). Mali Malaria Operation Plan FY 2013  UNOCHA (2012). Humanitarian Response COD-FOD registry. Accessed 04 November, 2013 from https://cod.humanitarianresponse.info/search/field_country_region/144  http://www.sante.gov.ml/  Personal communication Dr Ibrahim Socé Fall, WR-WHO, Mali |
| Mauritania | Moughataa  [**53**] | Ministère de la Sante (2011). Plan National de Développement Sanitaire 2012 -2020  Ministère de la Sante (2005). Plan Stratégique National de Lutte Contre les Epidémies de Paludisme, 2006–2010  http://www.sante.gov.mr/msas/index |
| Niger | District Sanitaire  [**42**] | Ministère de la Sante (2011). Plan Stratégique de Lutte Contre Le Paludisme 2011-2015  République du Niger (2002). Portant création des Communes et fixant le nom de leurs chefs-lieux: Loi n° 2002-014 du 11 Juin 2002 http://www.case.ibimet.cnr.it/den/Documents/code_rural‌/cdrom/doc%20pdf/Loi%20N%B02002-14%20cr%E9ation%20des%20communes.pdf  http://www.msp.ne/ |
| Nigeria (Northern states) | Local Government Authority  [**207**] | Federal Ministry of Health (2010). National Strategic Health Development Plan (NSHDP) 2010 - 2015  http://www.health.gov.ng/ |
| Senegal | District Sanitaire  [**76**] | President’s Malaria Initiative (2013). Senegal Malaria Operational Plan FY 2013  http://www.senegalaisement.com/senegal/decoupage_administratif_senegal.php  http://www.au-senegal.com  http://www.sante.gouv.sn/ |

**Section C: Population distribution mapping**

The countries of the sub-Sahel are characterised by an over-distributed pattern of human settlement governed by access to water, trade routes and proximity to urban centres. This over-dispersion of human hosts is a critical feature to capture at the highest possible spatial resolution for all vector-borne diseases.

Modelling techniques for the spatial reallocation of populations within census units have been developed in an attempt to (i) disaggregate population count data to a finer spatial detail and (ii) convert population count data from irregular administrative units to regular raster layers [5,6,7,8]. Human population census data, official population size estimates and corresponding census enumeration unit boundaries at the highest spatial resolution available from the most recent available censuses were acquired for each country. Table B summarizes the spatial resolution, year and source of all data used. Typical regional per-land cover class population densities were estimated from African countries for which very fine resolution population data were available, following approaches previously outlined [7]. These population densities were then applied as weightings to redistribute census counts according to the land cover and to map human population distributions at a finer spatial resolution. The modelling method distinguishes urban and rural populations in the redistribution of populations. Major settlements have population numbers already derived and validated and this makes up around 40% of the total population in the eight Sahelian countries [9]. The remaining 60% rural population was redistributed using land cover-based weightings and dasymetric modelling techniques [10].

The population datasets of the Sahelian countries were projected to 2015-2020 using national rural and urban growth rates estimated by the UN Population Division [11] using the following equation:

$$P_{y}=P_{d}e^{rt}$$

where *P_y_* is the population for year *y* within a pixel, *P_d_* is the population within the same pixel at the year of the input population data, *t* is the number of years between the input data and year *y*, and *r* is the average growth rate. Finally, datasets were adjusted to ensure that national population totals matched those reported by the UN [11].

Data on sub-national population age structures were obtained from a variety of sources (Table C) and used to derive adjustments for of population density by age categories: under five years, 5-9 years, 10-14 years and 15+ years. To compute the proportion of children below the age of 3 months to under five years, the estimates for children under the age of five years was discounted by the proportion of children < 3months from multiple indicator cluster surveys [12]. The datasets were then adjusted to ensure that national population totals by age group matched those reported by the UN [11]. The final datasets allowed for the extraction of populations for the years 2014 - 2015, with specific reference to gridded population counts of children aged 3-59 months and 3-119 months of age in 2015 - 2020.

The accuracy of the population distribution maps is largely determined by the spatial resolution and the currency of the census data [7]. All the countries in this study have had a census since 2009, except for Burkina Faso and Nigeria where the last censuses were done in 2006 (Table B and C). Despite the recent censuses, data used to develop the high resolution population maps were only available at the second administrative unit, the equivalent of a district, for Chad, the Gambia and Mauritania [13]. For Senegal, the 2012 census data are as yet unavailable and the current maps are based on projection from 2002.

**Table B** Summary of population data used by country

| **Country** | **Admin level used (level)**  **[Number of units]** | **Average spatial resolution ^a^** | **Average People per admin unit (1000's)** | **Last census year** | **Population data source** b |
| --- | --- | --- | --- | --- | --- |
| Burkina Faso | Department (3) [352] | 28 | 47 | 2006 | Institut National de la Statistique et de la Démographie (INSD) |
| Chad | Department (3) [62] | 144 | 181 | 2009 | Institut National de la Statistique, des Etudes Economiques et Démographiques (INSEED), Chad |
| Gambia | District  (2) [37] | 17 | 47 | 2003 | Gambia Bureau of Statistics |
| Mali | Commune  (4) [687] | 43 | 22 | 2009 | Institut National de la Statistique du Mali, Mali |
| Mauritania | Moughataa  (2) [45] | 152 | 77 | 2000 | Office National de Statistique (ONS), Mauritanie |
| Niger | Commune  (3) [260] | 68 | 60 | 2012 | Institut National de la Statistique, Niger |
| Nigeria | LGA  (2) [774] | 34 | 205 | 2006 | National Bureau of Statistics, Nigeria |
| Senegal | Commune  (4) [332] | 24 | 37 | 2002 ^c^ | Agence Nationale de la Statistique et de la Démographie, Sénégal |

a. The ASR measures the effective resolution of administrative units in kilometres. It is calculated as the square root of the land area divided by the number of administrative units

b. Most datasets have been downloaded from GeoHive, 2000-2013 [http://www.geohive.com/]

**Table C** Data sources used for the derivation of age proportions to generate the age-structured population surfaces

| **Country** | **Data type used** | **Year** | **Admin unit level** | **Data source** |
| --- | --- | --- | --- | --- |
| Burkina Faso | Census | 2006 | 1 | Institut National de la Statistique et de la Démographie (INSD) |
| Chad | DHS | 2004 | 1 | MEASURE Demographic and Health Surveys, USAID |
| Gambia | MICS | 2006 | 1 | UNICEF |
| Mali | Census microdata | 1998 | 2 | Integrated Public Use Microdata Series, International (IPUMSI) |
| Mauritania | MICS | 2007 | 1 | UNICEF |
| Niger | DHS | 2006 | 1 | MEASURE Demographic and Health Surveys, USAID |
| Nigeria | Census | 2006 | 1 | National Bureau of Statistics, Nigeria |
| Senegal | Census microdata | 2002 | 2 | IPUMSI |

**Section D: Identifying areas of acute seasonal transmission targets for SMC**

The approach developed by Cairns and colleagures [14] was used to define areas of acute seasonal transmission. In this approach the optimal model for malaria seasonality identified areas where 60% or more of the annual total rainfall occurs in three consecutive months. To replicate this approach, we used daily rainfall estimates from the Africa Rainfall Estimates version 2 (RFE 2.0) data from 2002-2009 at 10 × 10 km spatial resolution [15]. The RFE dataset was developed as a collaborative programme between NOAA’s Climate Prediction centre (CPC), USAID/Famine Early Systems Network (FEWS). The input data used for RFE2.0 rainfall estimates are obtained from 4 sources; 1) Daily Global Telecommunication Station (GTS) rain gauge data for up to 1000 stations which are then interpolated; 2) Advanced Microwave Sounding Unit (AMSU) microwave satellite precipitation estimates up to 4 times per day; 3) NOAA Special Sensor Microwave/ Imager (SSM/I) satellite rainfall estimates up to 4 times per day 4) GEOS Precipitation Index (GPI) cloud-top IR temperature precipitation estimates on a half-hour basis [15,16]

The daily surfaces were summed to generate monthly rainfall raster grid from January 2002 to December 2009. These data were used further to generate synoptic monthly average rainfall, which were then used to develop 12 three-month total average rainfall surfaces. These surfaces were then divided by the total average annual rainfall to compute the proportion of rainfall represented by each monthly grid. Areas where ≥60% of rainfall occurred within any three consecutive months were then selected as acutely seasonal. Analysis was done in ArcGIS (Version 10.1. ESRI Inc., USA).

**Section E: Computing the number of months of malaria transmission**

The numbers of SMC campaigns depend on the number of months in a transmission season. The WHO recommendation is that an SMC dose of 1 tablet of SP and 3 tablets AQ over three days are given to each during each month of the transmission season [17]. To compute the number of transmission months, a climate based model of months suitable for transmission was downloaded as a grid surface from International Research Institute for Climate and Society website [, 18]. This surface was developed from long-term rainfall and temperature data and their theoretical relationship with *P. falciparum* malaria transmission [19]. Areas where the monthly precipitation accumulation was at least 80 mm, the monthly mean temperature of between 18°C and 32°C and the monthly relative humidity of at least 60% were considered suitable for malaria transmission. Transmission suitability was defined at spatial resolution of approximately 50 x 50 km and was resampled to 1 x 1 km. This was extracted to each health district in ArcGIS 10.1 to compute the median number of transmission months.

**Section F: Mapping the intensity of *P. falciparum* transmission in 2000 and 2010**

Areas to be targeted for SMC have to have a moderate to high burden of malaria that is acutely seasonal. To role of the *P. falciparum* transmission maps in SMC was to inform the endemicity thresholds to define areas suitable for SMC and determine the age class of children to be targeted.

The procedures used to assemble, geo-code, archive, model and validate the transformation of empirical *P. falciparum* parasite prevalence data to continuous predictions of age-corrected mean prevalence in children aged 2-10 years (*Pf*PR_2-10_) are provided in Noor *et al* 2014 [1]. In brief, we have used information from available age-corrected survey data (sample size and numbers positive) at known locations (longitude and latitude) and times (year) with a minimal set of conservative, long-term covariates traditionally used in vector-borne disease mapping within a Bayesian hierarchical space-time model, implemented through an adapted Stochastic Partial Differential Equations (SPDE) approach using Integrated Nested Laplace Approximations (INLA) for inference [20,21] to produce continuous maps of *Pf*PR_2-10_ for 2000 and 2010 at 1 x 1 spatial resolutions [1]. A spatially and temporally de-clustered 10% of the *Pf*PR_2-10_ data was held out per country for model validation. Model accuracy was estimated by computing the linear correlation, the mean prediction error (MPE) and mean absolute prediction error (MAPE) of the observations and predictions of the holdout dataset. MPE is a measure of the bias of predictions (the overall tendency to over or under predict) whilst the MAPE is a measure of overall precision (the average magnitude of error in individual predictions.

The continuous *Pf*PR_2-10_ surfaces were used together with the 2000 and 2010 population surface by country at matching 1 × 1 km spatial resolutions to compute population adjusted *Pf*PR_2-10_ (PA*Pf*PR_2-10_) by health district. To do this, the mean posterior *Pf*PR_2-10_ (proportion) was multiplied with the pixel level population in 2000 and 2010 to generate an estimate of number of likely infected persons per pixel per year, which was the summed for each health district. This was then divided by the total population of the health district in each year to generate a mean PA*Pf*PR_2-10_.

**Section G: Identifying SMC suitable areas, computing population of target children and number of antimalarial tablets required**

For each health district the proportion of the population that was located in areas where ≥60% of rainfall occurred within any 3 consecutive months was computed. Then the continuous 2000 and 2010 *Pf*PR_2-10_ surface was used together with the population surface for corresponding years [8] at matching 1 × 1 km spatial resolutions to compute population adjusted 2000 and 2010 *Pf*PR_2-10_ (PA*Pf*PR_2-10_) by health district. To compute PA*Pf*PR_2-10_, the mean proportion of the posterior *Pf*PR_2-10_ was multiplied with the pixel level population surface to generate an estimate of number of people who were likely to be infected per pixel in 2000 and 2010, which was the summed for each health district. To generate the mean PA*Pf*PR_2-10_ the estimated population that was infected was divided by the total population of the health districts in 2000 and 2010. Health districts were identified as suitable for SMC if they were seasonal (>80% of population lived in areas where >=60% of rainfall occurred in 3 consecutive months) and had 2000 PA*Pf*PR_2-10_ (receptive risk) of ≥5%.

Districts identified as SMC targets were further classified into areas were _2000_PA*Pf*PR_2-10_ ≥5% to ≤10% to target children aged 3 months to 10 years; and _2000_PA*Pf*PR_2-10_ >10% to target children aged 3 months to 5 years based on the 2010 _2000_PA*Pf*PR_2-10_. This selection was guided by the expected age-dependency of immunity, infection and clinical disease to malaria transmission intensity. Under conditions of hypoendemic transmission (≤10% PA*Pf*PR_2-10_) older children are also susceptible to disease due to the low levels of exposure and subsequent delayed acquisition of immunity [25] and therefore are equally likely to benefit from SMC. Estimates of the overall and rural target population of children for 2015 to 2020 were then extracted for each health district from the population raster surfaces in ArcGIS 10.1.

A sensitivity analysis of the population seasonality threshold was implemented at 40%, 50%, 60% and 80% of population living in acutely seasonal areas (Fig. C). There was a difference of 29 districts or 1.2 million children 3m to <5 years between a 20% and 80% seasonality + population thresholds. The contributors to the additional districts were Nigeria (15), Chad (7), Burkina Faso (30 and Mali (3). Given the limited gain in population coverage relative to the likely increase in operational costs in visiting the additional districts where only a small proportion need SMC, we have elected to use the 80% threshold.

**Fig. C** The number of I) districts; II) children 3m to 5 years to be targeted for SMC at different thresholds of population within a district leaving in areas classified as having acute malaria seasonality and have mean PAPfPR_2-10_ of >5%.

I)

**
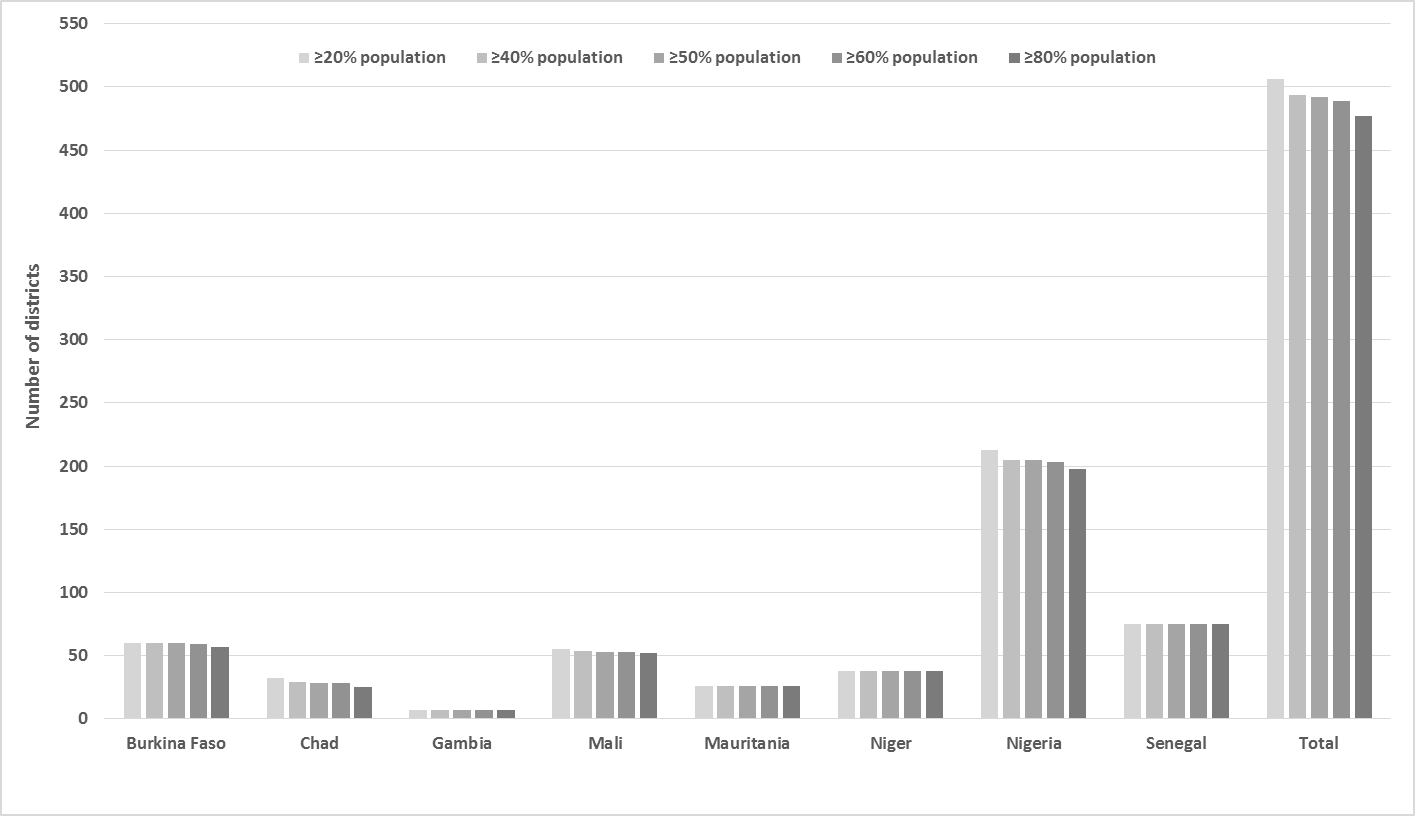
**

II)


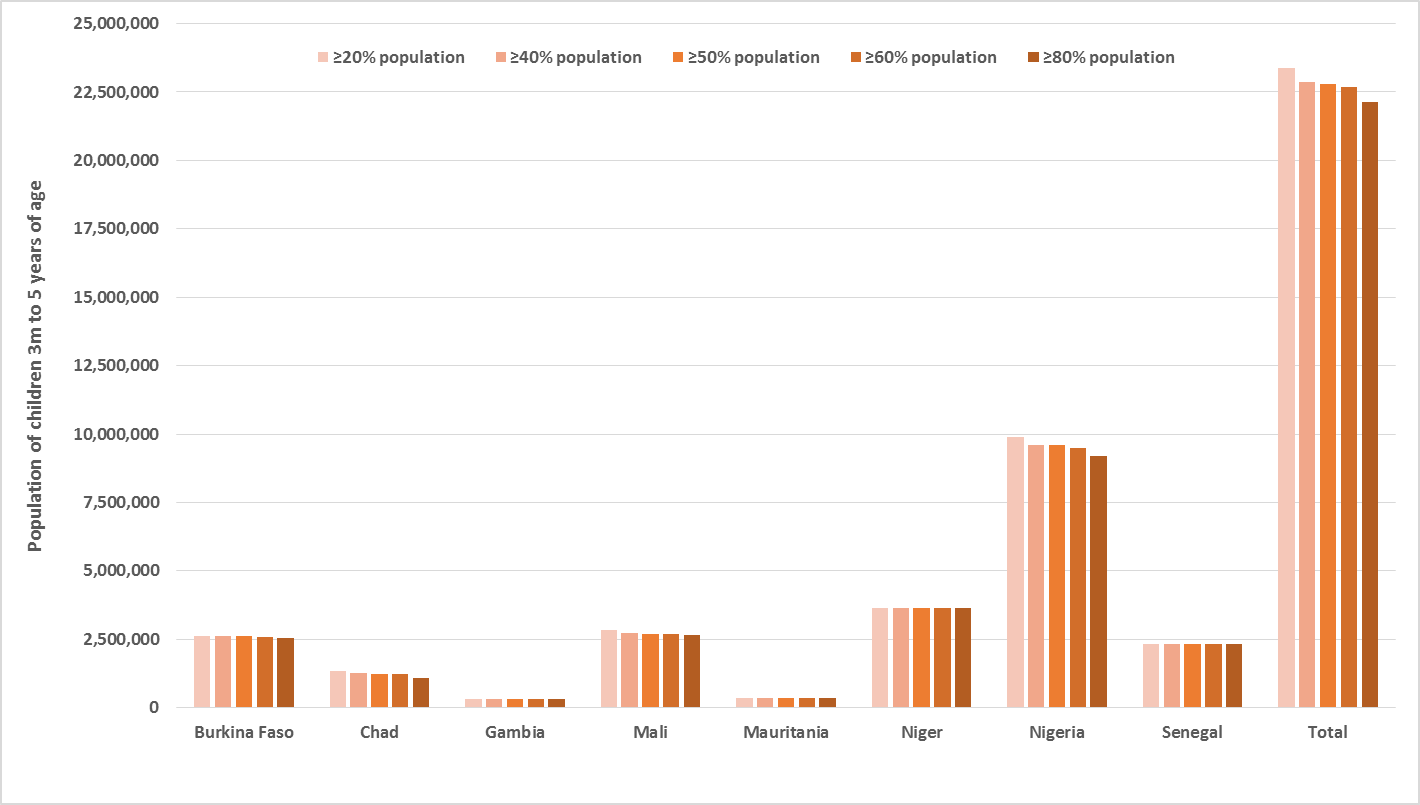


For the year 2015, the estimated number of children to be targeted for SMC was then multiplied by the number of malaria transmission months per health district (SI 1.5) and the SP and AQ doses required per child per month of 1 dose of SP and AQ on day 1 and one dose of AQ in each of day 2 and 3 [18].

**References**

1. Noor AM, Kinyoki DK, Mundia CW, Kabaria CW, Wambua JM, Alegana VA, Fall IS, Snow RW (2014). The changing risk of Plasmodium falciparum malaria infection in Africa: 2000 to 2010. Lancet 2014; 383: 1739-1747.
2. Moonen B, Cohen JM, Snow RW, Slutsker L, Drakeley C, et al. Operational strategies to achieve and maintain malaria elimination. Lancet 2010; 376: 1592-1603
3. Global Administrative Unit Layers. Borders between countries and administrative units within the countries. Available: http://www.fao.org/geonetwork/srv/en/metadata.show?id=12691.
4. Second Administrative Level Boundaries. Available: http://salbgeonetwork.grid.unep.ch/geonetwork/srv/en/main.home
5. Tatem AJ, Noor AM, von Hagen C, Di Gregorio A, Hay SI. High resolution population maps for low income nations: combining land cover and census in East Africa. PLoS One 2007; 2: e1298
6. Linard C, Gilbert M, Tatem AJ. Assessing the use of global land cover data for guiding large area population distribution modelling. GeoJournal 2010; 76: 525–538
7. Linard C, Gilbert M, Snow RW, Noor AM, Tatem AJ. Population distribution, settlement Patterns and accessibility across Africa in 2010. PLoS One2012; 7: e31743
8. World Population Datasets. Available: http://www.worldpop.org.uk/
9. Center for International Earth Science Information Network. Available: http://www.ciesin.org/
10. Mennis J. Dasymetric mapping for estimating population in small areas. Geography Compass 2009; 3: 727-745
11. United Nations Population Division (2012) World Population Prospects: The 2012 Revision (United Nations, New York). Available: http://esa.un.org/wpp/
12. Multiple Indicator Cluster Surveys. Available: http://mics.unicef.org/surveys
13. Geohive. Available: http://www.geohive.com/
14. Cairns M, Roca-Feltrer A, Garske T, Wilson AL, Diallo D, Milligan PJ, et al. Estimating the potential public health impact of seasonal malaria chemoprevention in African children. Nature Comms 2012; 3: 881.
15. NOAA (2013). The NOAA Climate Prediction Center: African Rainfall Estimation AlgorithmVersion2.0. Available: from http://www.cpc.ncep.noaa.gov/products/fews/RFE2.0_tech.pdf on 09/01/2014.
16. Novella N, Thiawa W. Africa rainfall climatology version2, 2012. NOAA/Climate Prediction Center. Available: www.cpc.ncep.noaa.gov/products/fews/AFR_CLIM/afr_clim
17. World Health Organization. Seasonal Malaria Chemoprevention with sulphadoxine-pyrimethamine and amodiaquine in children: a field guide. World Health Organization, July 2013. Available: http://apps.who.int/iris/bitstream/10665/85726/1/9789241504737_eng.pdf.
18. Seasonal Climatic Suitability for Malaria Transmission. Available: http://iridl.ldeo.columbia.edu/maproom/Health/Regional/Africa/Malaria/CSMT/index.html.
19. Grover-Kopec EK, Blumenthal MB, Ceccato P, Dinku T, Omumbo JA, et al. Web-based climate information resources for malaria control in Africa. Malar J 2006; 5:38.
20. R-INLA project. Available: http://www.r-inla.org/
21. Rue H, Martino S, Chopin N. Approximate Bayesian inference for latent Gaussian models by using integrated nested Laplace approximations. J. R. Stat. Soc 2009; 71: 319–392
22. Gething PW, Van Boeckel, Smith DL, Guerra CA, Patil AP, Snow RW, Hay SI. Modelling the global constraints of temperature on transmission of Plasmodium falciparum and P. vivax. Parasites & Vectors 2011; 4: 92
23. Scharlemann J, Benz D, Hay S, Purse B, Tatem A, et al. Global data for ecology and epidemiology: a novel algorithm for temporal Fourier processing MODIS data. PLoS One 2008; 9:e1408.
24. Hijmans R, Cameron S, Parra J, Jones P, Jarvis A. Very high resolution interpolated climate surfaces for global land areas. Int. J. Climatol 2005; 25: 1965-1978
25. Snow RW. Global malaria eradication and the importance of Plasmodium falciparum epidemiology in Africa. BMC Med 2015; 13:23. doi: 10.1186/s12916-014-0254-7.
